# Which treatment is preferred for advanced non-small-cell lung cancer with wild-type epidermal growth factor receptor in second-line therapy? A meta-analysis comparing immune checkpoint inhibitor, tyrosine kinase inhibitor and chemotherapy

**DOI:** 10.18632/oncotarget.20281

**Published:** 2017-08-16

**Authors:** Di Wu, Chongyang Duan, Fenfang Wu, Liyong Chen, Size Chen

**Affiliations:** ^1^ Central Laboratory, The First Affiliated Hospital of Guangdong Pharmaceutical University, Guangzhou, China; ^2^ Department of Biostatistics, School of Public Health, Southern Medical University, Guangzhou, China; ^3^ Guangdong Province Key Laboratory for Medical Molecular Diagnostics, China-America Cancer Research Institute, Dongguan Scientific Research Center, Guangdong Medical University, Dongguan, China

**Keywords:** immune checkpoint inhibitor, tyrosine kinase inhibitor, chemotherapy, wild-type epidermal growth factor receptor, lung cancer

## Abstract

**Background:**

The recommendations regarding the optimum treatment for advanced non-small-cell lung cancer (NSCLC) patients with wild-type (WT) epidermal growth factor receptor (EGFR) tumors remain unclear. This meta-analysis was conducted to assess the efficacy among programmed death-ligand 1 (PD-L1)/programmed death-1 (PD-1) antibody, EGFR-tyrosine kinase inhibitors (TKI) and chemotherapy in second-and third-line therapy.

**Patients and methods:**

Randomized trials investigating two of the three treatments were searched and included. Multiple treatments comparison and pairwise comparison were performed to assess overall survival (OS) and progression-free survival (PFS), expressed as hazard ratios (HRs). The effect of prespecified study-level characteristics was assessed by subgroup analysis and meta-regression.

**Results:**

12 randomized trials accruing 3341 advanced patients with WT EGFR tumors were analyzed. PD-1/PD-L1 antibody was associated with significantly longer OS and PFS than chemotherapy (OS: HR 0.67, 95% CrI 0.60–0.75; PFS: HR 0.83, 95% CrI 0.73–0.95) and TKI (OS: HR 0.59, 95% CrI 0.50–0.70; PFS: HR 0.75, 95% CrI 0.66–0.84) , while chemotherapy was associated with significantly longer OS (HR 0.88, 95% CrI 0.77–0.99) and PFS (HR 0.75, 95% CrI 0.66–0.84) than TKI.

**Conclusions:**

For advanced NSCLC patients with WT-EGFR tumors in second- or third-line therapy, PD-1/PD-L1 antibody appeared to be the most efficacious treatment, which was followed by chemotherapy. EGFR-TKI was worse than chemotherapy.

## INTRODUCTION

Worldwide, lung cancer is the leading cause of cancer mortality, making up about 19% of all cancer-related deaths [1]. Most of cases (∼85%) are histologically defined as non-small-cell lung cancer (NSCLC) [2]. Of these patients, nearly two-thirds present with unresectable locally advanced or metastatic disease at initial diagnosis.[3] Since the discovery that epidermal growth factor receptor (EGFR)-sensitizing mutations are frequent oncogenic driver of NSCLC, many randomized trials were performed and demonstrated the benefit of EGFR tyrosine kinase inhibitors (TKI) over cytotoxic chemotherapy in patients with EGFR-sensitizing tumors [4-11]. EGFR-TKI is thus recommended as standard first-line treatment for this drug-sensitive subgroup [12]. However, these mutations occur in only about 15% of White and Africa America patients and mostly 50% of Asian patients. Most of patients worldwide have wild-type (WT) EGFR tumors and the recommendations regarding the optimum treatment for these patients still remain elusive [12, 13].

First-generation EGFR-TKI (ie., gefitinib and erlotinib), which act through reversibly binding to and blocking EGFR signaling pathway, have also been widely used in NSCLC patients with WT EGFR tumors [14]. Although the landmark trial BR21 showed that erlotinib significantly extended both overall survival (OS) and progress-free survival (PFS) over placebo in EGFR-unselected, pretreated patients [15], the superiority of EGFR-TKI over platinum-based chemotherapy was less pronounced. Recent randomized trials [4, 16-19] and meta-analyses [20, 21] showed that TKI was associated with shorter PFS than chemotherapy in this subpopulation. However, OS is commonly considered a more important clinical endpoint than PFS [22]. Till now, except TAILOR trial, no studies had identified a survival improvement, which largely attribute to treatment crossover after progression and small sample size included in individual trials.

With profoundly different cure mechanism from TKI or chemotherapy, immune checkpoint inhibition therapy aims to enhance an effective immune response through restoring the efficacy of tumor-specific T cells within the tumor microenvironment and has shown promising outcomes in different cancers, including NSCLC [23]. Recent randomized trials showed that, programmed death-ligand 1 (PD-L1) and programmed death-1 (PD-1) antibodies, two kinds of checkpoint inhibitors which promote anticancer immunity by blocking PD-1 and PD-L1 interaction and reactivate suppressed immune cells, significantly extended survival over chemotherapy in second- and third-line therapy in advanced NSCLC patients, including the WT EGFR patients [24-27].

In this meta-analysis of randomized trials, we compared the efficacy of PD-1/PD-L1 antibody, first-generation EGFR-TKI and chemotherapy in second- or third-line setting with Bayesian indirect method that allowed for combining direct and indirect evidence, aiming to identify the optimum treatment that could provide best survival benefit for advanced NSCLC patients with WT EGFR tumors.

## RESULTS

### Characteristics of included trials and patients

**Figure 1:**
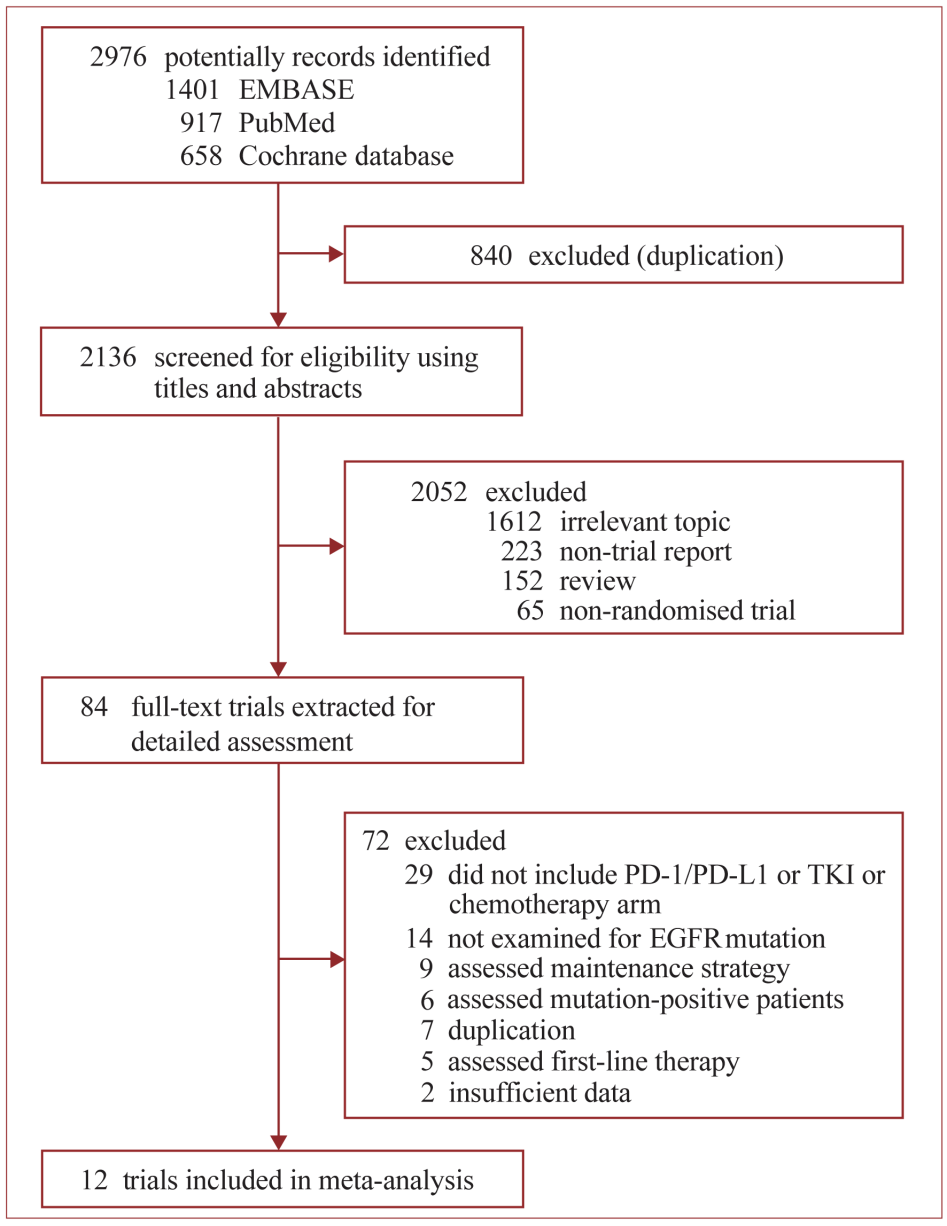
Identification of eligible randomized trials Abbreviations: EGFR, tyrosine kinase inhibitors; PD-1, programmed death-1; PD-L1, programmed death-ligand 1; TKI, tyrosine kinase inhibitors.

Of 2976 potentially records were initially identified by search strategy (Figure 1), 12 open-labeled, randomized Phase II/III trials accruing 6462 patients with advanced NSCLC were finally included in this meta-analysis (Table 1) [17-19, 24-33]. After excluding the patients who did not have a known EGFR mutation status, a total of 3341 patients bearing WT EGFR tumors were included. All trials enrolled patients aged > 18 years and had a histologically or cytologically confirmed diagnosis of NSCLC, with Eastern Cooperative Oncology Group or World Health Organization performance status of 0 to 2. All the four trials containing PD-1/PD-L1 antibody arm used FDA-approved dose. Three of them were performed in second- or third-line setting [24, 25, 27], the other one were second- setting [26]. All 12 trials containing chemotherapy arm used recommended drugs (single-agent docetaxel or pemetrexed is standard second- or higher- line treatment [12, 13]) with standard dosing schedule. All the 8 trials containing EGFR-TKI arm used standard dosing schedule (erlotinib, 150 mg orally daily; gefitinib, 250 mg orally daily). Among these trials, five were second-line setting [17, 19, 30, 32, 33], and three were second- or third-line setting [18, 28, 29, 31]. Five trials majorly comprised of white patients [17, 28-31, 33], while the other three majorly included Asian patients [18, 19, 32]. Four trials used only direct sequencing for EGFR mutation detection [19, 28-31], while the other four used more sensitive methods (amplification refractory mutation system, restriction fragment length polymorphism analysis, highly sensitive polymerase chain reaction or mass spectrometry) to enhance sensitivity [17, 18, 32, 33].

**Table 1:** List of Trials Characteristics

Source	Design	Entry Criteria	Line	Dominant Ethnicity, No. (%)	Age (years)	EGFR Mutation testing	Treatments and dosing schedule	Arm A	Arm B	Median follow-up (mon)
								**WT/Total**	**WT/Total**	
PD-1/PD-L1 vs CT										
CheckMate 057, 2015	Open label, International, multicenter, phase 3	Patients with advanced nonsquamous NSCLC that had progressed during or after platinum-based CT PD-L1 positive	Second or third	White (91)	62 (21–85)	NR	Nivolumab (3 mg/kg every two weeks) vs docetaxel (75 mg/m2 every three weeks)	168/292	172/290	NR
KEYNOTE-010, 2015	Open label, International, multicenter, phase 2/3	Patients with advanced NSCLC that had progressed after platinum-based CT or TKI	Second	non-Asian (82)	62 (56–69)	Direct Sequencing/ ARMS	Pembrolizumab (2 mg/kg or 10 mg/kg every three weeks) vs docetaxel (75 mg/m2 every three weeks)	581/ (344/346)	294/343	13.1 (IQR 8.6–17.7).
POPLAR, 2016,	Open label, International, multicenter, phase 2	Patients with advanced NSCLC that had progressed after platinum-based CT or TKI	Second or third	White (100)	62 (36–84)	NR	Atezolizumab (1200 mg fixed dose every 3 weeks) vs docetaxel (75 mg/m2 every three weeks)	147/287		14.8 vs 15.7* (0.1–19.6)
OAK, 2016	Open label, International, multicenter, phase 3	Patients with advanced NSCLC that had received platinum-based CT	Second or third	White (70)	64 (33–85)	Direct Sequencing/ ARMS	Atezolizumab (1200 mg fixed dose every 3 weeks) vs docetaxel (75 mg/m2 every three weeks)	318/613	310/612	21.0 (NR)
TKI vs CT										
INTEREST 2008 and 2010	Open label, International, multicenter, phase 3	TKI-naïve patients with advanced NSCLC that had progressed or recurred after platinum-based CT	Second or third	White (74.4)	61 (20–84)	Direct sequencing	Gefitinib (250 mg per day orally) vs docetaxel (75 mg/m2 every three weeks)	123/733	106/733	7.6 (NR)
TITAN, 2012	Open label, International, multicenter, phase 3	TKI and pemetrexed-naïve patients with advanced NSCLC that had progression during or after platinum-based CT	Second	White (84.5)	59 (22–80)	Direct sequencing	Erlotinib (150 mg per day orally) vs docetaxel (75 mg/m2 every three weeks) or pemetrexed (500 mg/m2 every three weeks)	74/221	75/203	27.9 vs 24.8* (0.0–50.3)
TAILOR, 2013	Open label, multicenter, phase 3	TKI And taxanes-naïve patients with advanced NSCLC that had recurred or progressed after CT	Second	White (99.1)	67 (35–83)	Direct sequencing + fragment analysis	Erlotinib (150 mg per day orally) vs docetaxel (75 mg/m2 every three weeks)	110/110	109/112	33.0 (NR)
CT/06.05, 2013	Open label, multicenter, phase 3	TKI and pemetrexed-naïve patients with advanced NSCLC that had progressed during after CT	Second or third	White (NR)	66 (37–86)	Direct sequencing	Erlotinib (150 mg per day orally) vs pemetrexed (500 mg/m2 every three weeks)	57/178	55/179	29.0 vs 27.3* (NR)
NCT01565538, 2014	Open label, phase 2	TKI and pemetrexed-naïve patients with advanced NSCLC that had progressed during or after CT	Second	Asian (NR)	55 (30–75)	ARMS + FISH	Erlotinib (150 mg per day orally) vs pemetrexed (500 mg/m2 every three weeks)	62/62	61/61	14·7 (0.5–41.9)
PROSE, 2014	Open label, multicenter, phase 3	TKI-naive patients with advanced NSCLC that that had recurred or progressed during or after CT	Second	White (NR)	66 (33–85)	MS	Erlotinib (150 mg per day orally) vs docetaxel (60 mg/m2 every three weeks) or pemetrexed (500 mg/m2 every three weeks)	82/143	81/142	32·4 (IQR 22.3–44.5)
DELTA, 2014	Open label, multicenter, phase 3	TKI and docetaxel-naïve patients with advanced NSCLC that had progressed during or after platinum-based CT	Second or third	Asian (NR)	67 (31–85)	Highly sensitive PCR-based method	Erlotinib (150 mg per day orally) vs docetaxel (60 mg/m2 every three weeks)	90/151	109/150	8.9 (NR)
CTONG 0806, 2014	Open label, multicenter, phase 2	TKI and pemetrexed-naïve patients with advanced NSCLC that had progressed after platinum-based CT	Second	Asian (NR)	57 (24–78)	Direct sequencing	Gefitinib (250 mg per day orally) vs pemetrexed (500 mg/m2 every three weeks)	76/76	81/81	10.6 (NR)

Abbreviations: ARMS, amplification-refractory mutation system; CT, chemotherapy; EGFR, epidermal growth factor receptor; FISH, fluorescence in situ hybridization; IQR, interquartile range; MS, mass spectrometry; NR, not reported; NSCLC; non-small-cell lung cancer; PD-1, programmed death-1; PCR, polymerase chain reaction; PD-L1, programmed death-ligand 1; TKI, tyrosine kinase inhibitor; WT, wild type.

*arm A vs arm B.

### Risk of bias assessment

The included trials were overall low risk (Table 2). Sequence was adequately generated in all trials. Allocation concealment was adequately performed in nine trials, not detailed in one trials [32] and undone in two trials [25, 27]. Though all trials were designed as open-labeled, six of them blinded assessment of outcome by independent, central radiologic reviews [24-27, 31] or independent review committee [19]. The reasons for excluding patients in all trials were sufficient and ITT principle was followed. No evidence of selective reporting was found. Additionally, other source of bias was found in two trials: one were halted prematurely [30], two had biased baseline characteristics [19], and the other one had imbalanced number of patients underwent crossover [32].

**Table 2:** The Assessment of Bias of Included Trials

Source	Sequence generation	Allocation concealment	Blinding	Incomplete data addressed	Selective reporting	Other source of bias
INTERIST, 2008 and 2010	Low risk (Minimization)	Low risk (Central allocation)	Unclear risk	Low risk	Low risk	Low risk
TITAN, 2012	Low risk (Minimization)	Low risk (Central allocation)	Unclear risk	Low risk	Low risk	Halted prematurely because of slow recruitment
TAILOR, 2013	Low risk (Minimization)	Low risk (Central allocation)	Unclear risk	Low risk	Low risk	Low risk
CT/06.05, 2013	Low risk (Randomized numbers generated by computer)	Low risk (Central allocation)	Low risk (Central radiologic review)	Low risk	Low risk	Low risk
NCT01565538, 2014	Low risk (Randomized numbers generated by computer)	Unclear risk	Unclear risk	Low risk	Low risk	61% patients in pemetrexed arm crossed over to erlotinib, while 10% patients in erlotinib arm crossed over to pemetrexed.
PROSE, 2014	Low risk (Minimization)	Low risk risk (Central allocation)	Unclear risk	Low risk	Low risk	Low risk
DELTA, 2014	Low risk (Minimization)	Low risk (Central allocation)	Unclear risk	Low risk	Low risk	Low risk
CTONG 0806, 2014	Low risk (Minimization)	Low risk risk (Central allocation)	Low risk (Independent Review Committee )	Low risk	Low risk	More nonsmokers in CT arm (57.9% vs 40.7%, p = 0.03)
CheckMate 057, 2015	Low risk (Minimization)	Low risk (Central allocation)	Low risk (Central radiologic review)	Low risk	Low risk	Low risk
KEYNOTE-010, 2015	Low risk (Randomized numbers generated by computer)	Low risk (Central allocation)	Low risk (Central radiologic review)	Low risk	Low risk	Low risk
POPLAR, 2016	Low risk (Randomized numbers generated by bracket)	High risk (undone)	Low risk (Central radiologic review)	Low risk	Low risk	Low risk
OAK, 2016	Low risk (Minimization)	High risk (undone)	Low risk ( Central radiologic review)	Low risk	Low risk	Low risk

### Standard pairwise comparison

**Figure 2:**
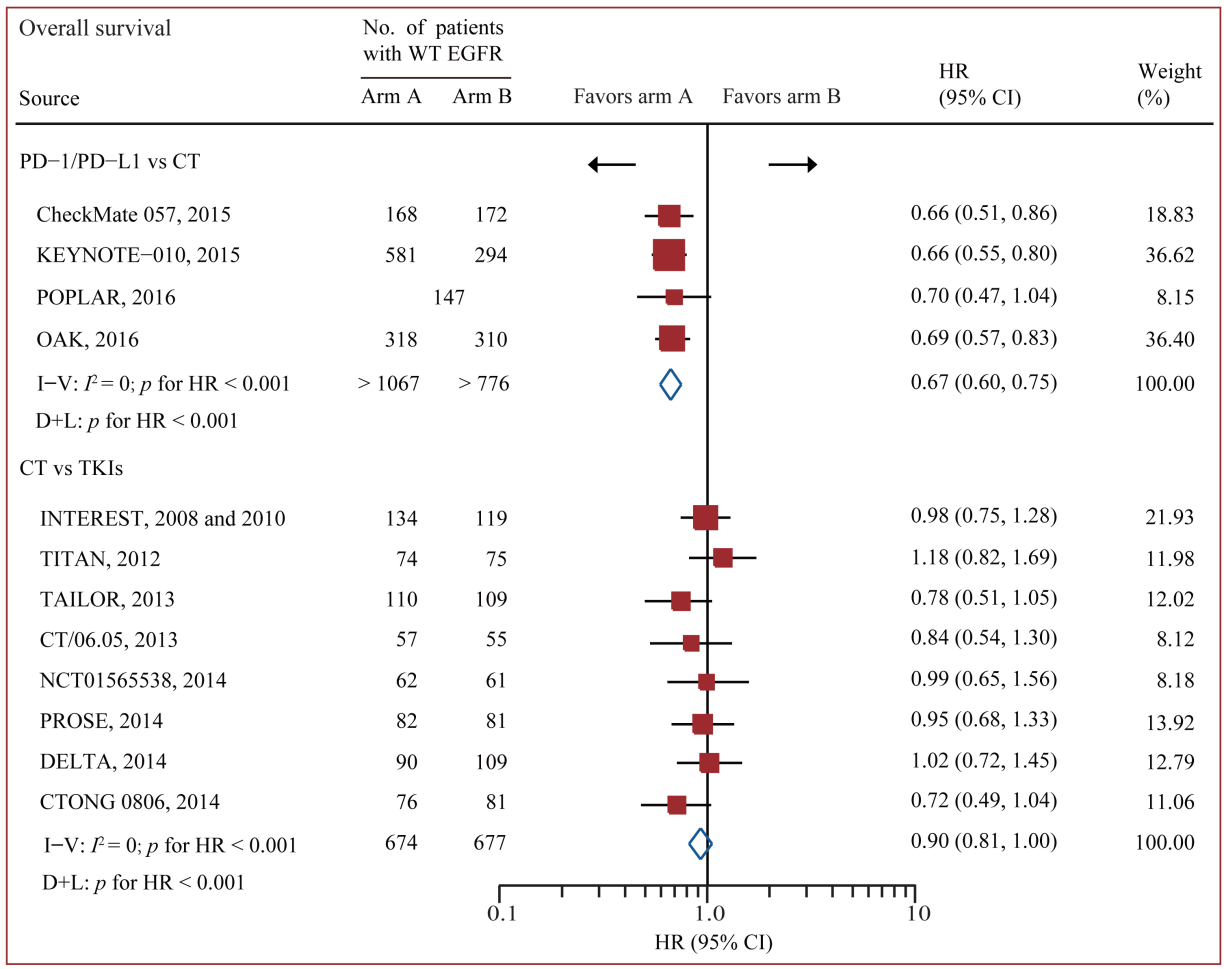
Pairwise comparisons for overall survival Abbreviations: CI, confidence interval; CT, chemotherapy; EGFR, epidermal growth factor receptor; HR, hazard ratios; I-V = inverse variance. D+L = DerSimonan and Laird; PD-1, programmed death-1; PD-L1, programmed death-ligand 1; TKI, tyrosine kinase inhibitors; WT, wild-type.

**Figure 3:**
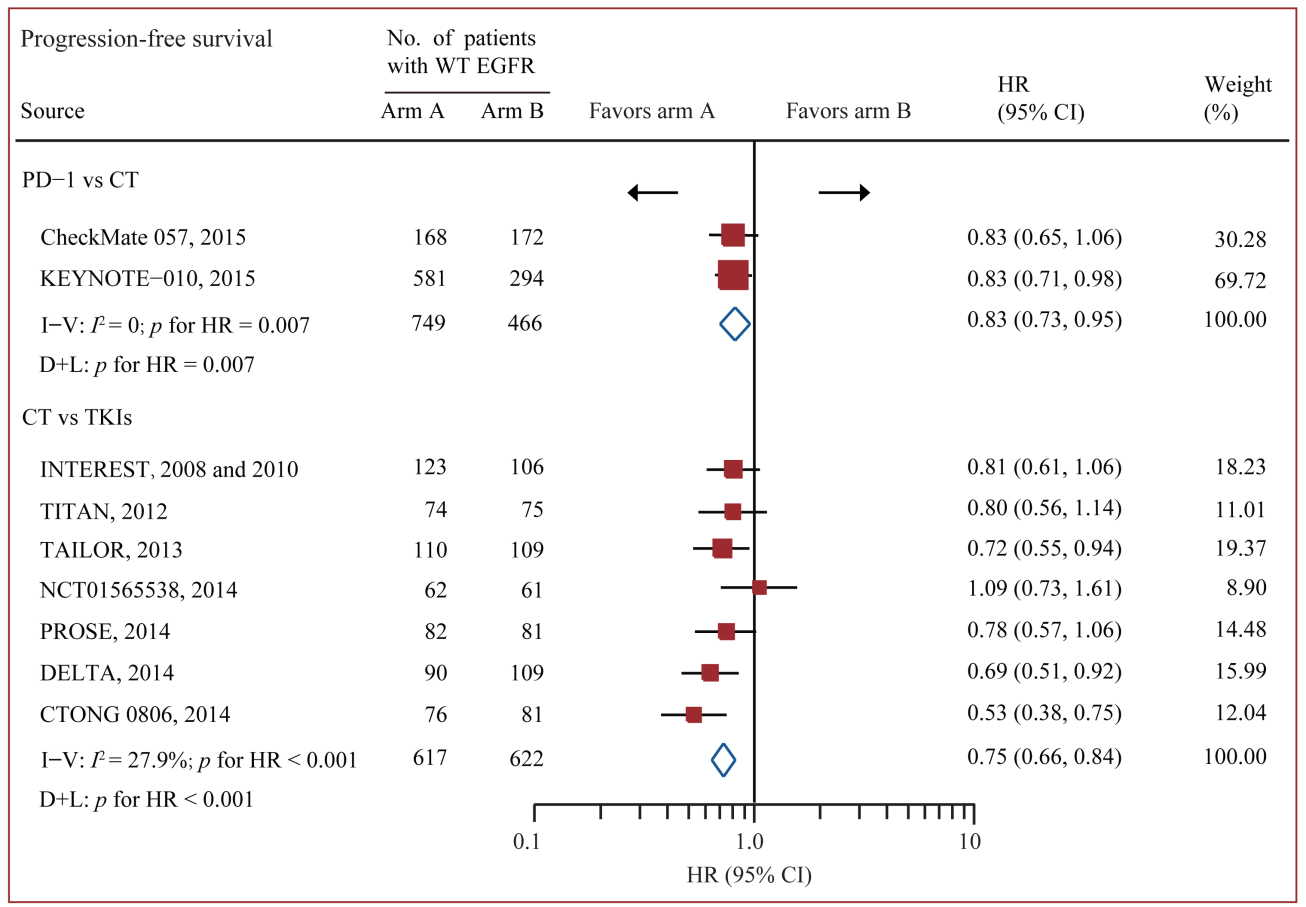
Pairwise comparisons for progression-free survival Abbreviations: CI, confidence interval; CT, chemotherapy; WT EGFR, epidermal growth factor receptor; HR, hazard ratios; I-V = inverse variance. D+L = DerSimonan and Laird; PD-1, programmed death-1; PD-L1, programmed death-ligand 1; TKI, tyrosine kinase inhibitors; WT, wild-type.

Data on OS were available from all 12 trials accruing 3341 patients [17-19, 24-33]. In standard pairwise comparisons (Figure 2 and Figure 3), no evidence of significant interstudy heterogeneity for OS or PFS was identified (*I*^2^ = 0% and 27%, respectively). The pooled fixed-effect models showed that treatment of PD-1/PD-L1 antibody was more effective in improving OS and PFS than chemotherapy in WT EGFR patients, with an estimated HR of 0.67 (95% CI 0.60-0.75, *p* < 0.001), and no significant difference for OS was identified between chemotherapy and EGFR-TKI. PFS was based on 9 out of 12 trials accruing 2454 patients.[17-19, 24, 26, 28-30, 32, 33] Treatment of PD-1 antibody significantly improved PFS compared with chemotherapy (HR 0.83 95% CI 0.73-0.95, *p* = 0.007), while treatment of chemotherapy significantly improved PFS compared with TKI (HR 0.75 95% CI 0.66-0.84, *p* < 0.001).

### Indirect comparison

**Figure 4:**
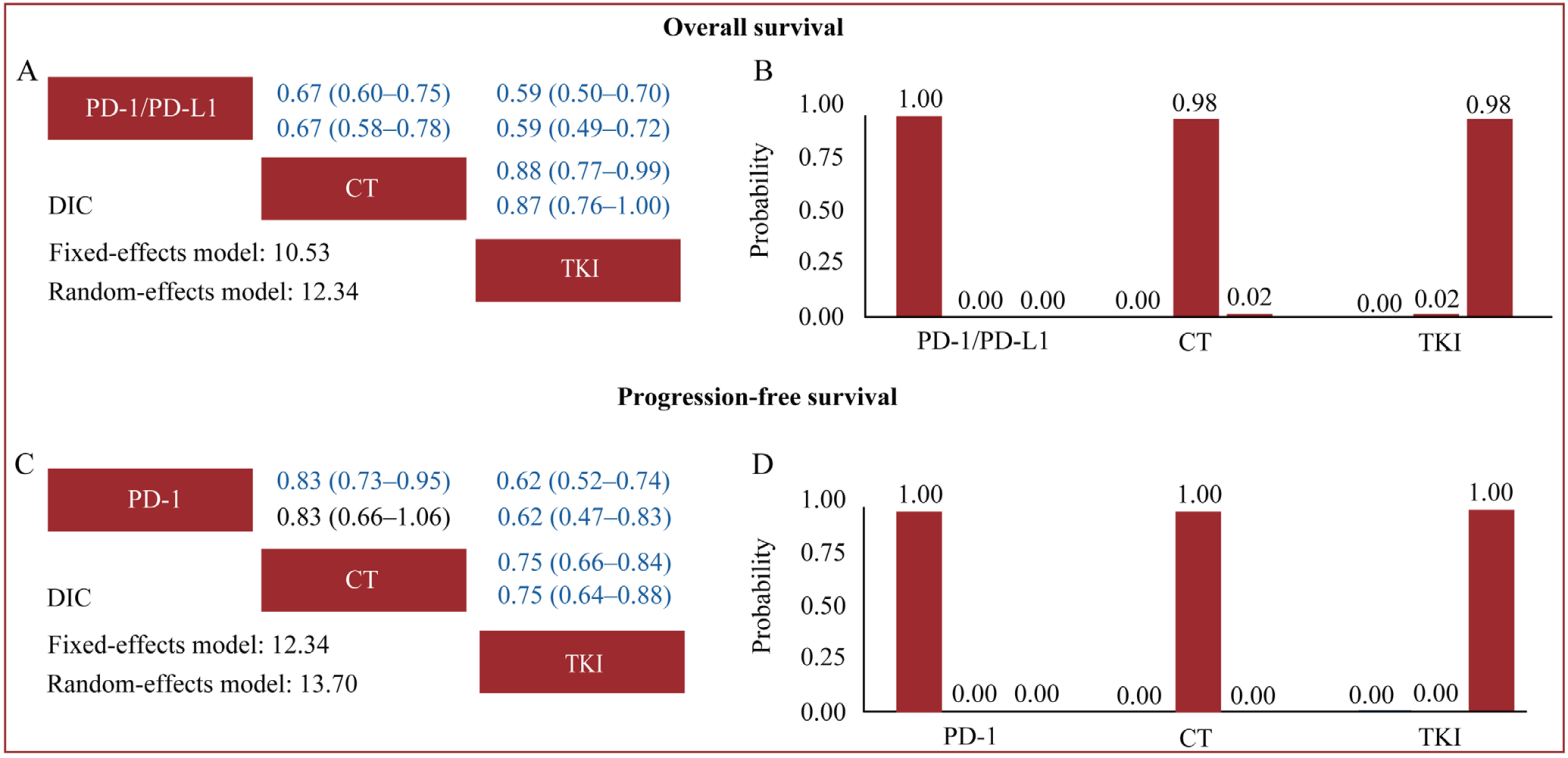
Indirect comparisons for overall survival **A.** and **B.** and progression-free survival **C.** and **D.** The row treatment was compared with column treatment; Upper triangles (A and C) denote pooled hazard ratios (HRs) with 95% credible intervals; In each cell, the first and second line used fixed-effects and random-effects models; HRs with Bayesian *p* value < 0.05 are in blue. Histograms (B and D) are shown for cumulative probabilities of each treatment ranking first, second and third best based on fixed-effects models. Abbreviations: CT, chemotherapy; DIC, deviance information criterion; EGFR, epidermal growth factor receptor; PD-1, programmed death-1; PD-L1, programmed death-ligand 1; TKI, tyrosine kinase inhibitors; WT, wild-type.

Based on the DIC values in indirect comparisons (Figure 4), the fixed-effects model had better model fit than random-effects models, with relatively lower DIC values for the two outcomes, suggesting that the interstudy heterogeneity might not be significant. We thus applied fixed-effects models in indirect comparisons. Pooled fixed-effects models showed that PD-1/PD-L1 antibodies were associated with significantly improved OS and PFS when compared with chemotherapy (cumulative OS: HR 0.67, 95% CrI 0.60-0.75; PFS: HR 0.83, 95% CrI 0.73-0.95) and TKI (cumulative OS: HR 0.59, 95% CrI 0.50-0.70; PFS: HR 0.75, 95% CrI 0.66-0.84) in patients bearing WT EGFR tumors, while chemotherapy was associated with significantly extended OS and PFS when compared with TKI (cumulative OS: HR 0.88, 95% CrI 0.77-0.99; PFS: HR 0.75, 95% CrI 0.66-0.84). Treatment rankings clearly showed that PD-1/PD-L1 antibodies had the highest probability (100%) of being the most effective treatment for both OS and PFS, which was followed by chemotherapy.

### Subgroup analysis

**Figure 5:**
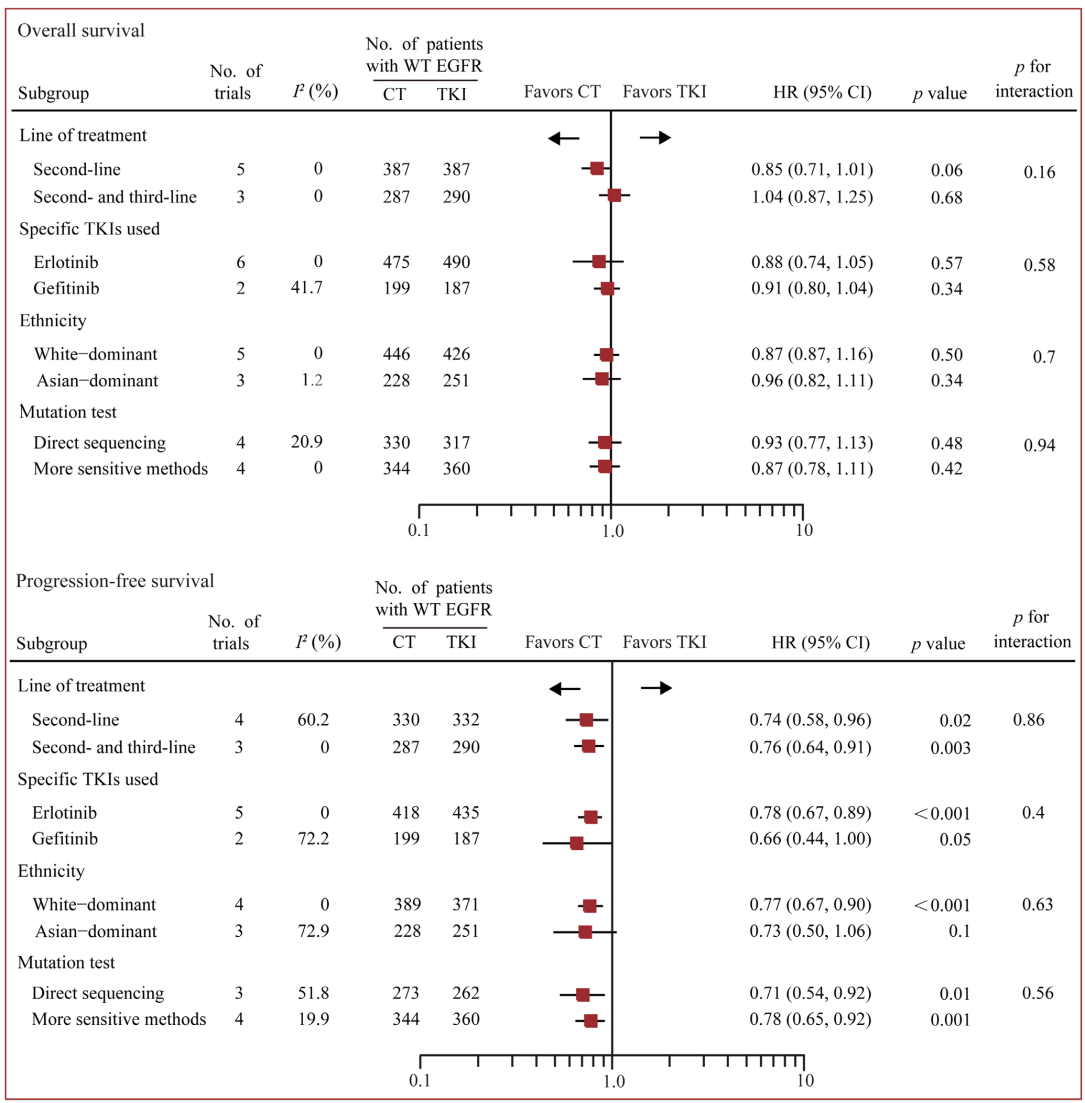
Subgroup analysis based on line of treatment, specific TKI used, ethnicity and method of EGFR mutation detection Fixed-effects models were used when *I*^2^ < 50%, otherwise random-effects models were used. CT, chemotherapy; EGFR, epidermal growth factor receptor; TKI, tyrosine kinase inhibitors; WT, wild-type.

Predefined multiple subgroups analysis and meta-regression was conducted to examine whether dominant ethnicity, line of treatment, the specific TKI used, or method of EGFR mutation detection was associated with overall treatment effects (Figure 5). In OS analysis, no statistically significant difference was detected in all these subgroups. Yet, there was a trend to favor chemotherapy than TKI in second-line setting, though the *p* value did not reach a significance threshold (HR 0.85, 95% CI 0.71-1.01, *p* = 0.06). In PFS analysis, chemotherapy was associated with longer PFS benefit than TKI in all subgroups, apart from the group of Asian patients.

## DISCUSSION

Current recommanded treatment for advanced NSCLC in first- and higher- line setting are based on the presence of genetic aberrations, such as the strong recommandation of EGFR-TKIs for pateints bearing EGFR sensitizing mutations. However, it should be realized that most NSCLC patients do not harbor these oncogenic drivers. For patients with WT EGFR tumors that account for a majority of NSCLC worldwide, the options were limited to cytotoxic chemotherapy in first-line setting, which being modest in extending survival; and in second- or higher line setting, the recommendation was less clear [13].

Enhancing the immune system to eliminate cancer cells is an effective way to prolong survival and time to progression. In contrast to disease-modifying agents such as cytotoxic chemotherapy and mutation-targeted drugs, PD-1/PD-L1 antibody unleashs suppressed T cell-mediated antitumor responses of the host by disturbing the PD-1 and PD-L1 interaction, showing promising effects in second- and third-line therapy in recent RCTs. Our pooled analysis showed that, for pre-treated WT EGFR patients, treatment of PD-1/PD-L1 antibody was more efficacious in prolonging survival compared with chemotherapy and EGFR-TKIs. For a median OS of 9.4 months in patients with standard-of-care docetaxel [24], the corresponding survival prolongation with PD-1/PD-L1 antibodies would be 3.1 months. Moreover, an improved secondary outcome of PFS was also identified in PD-1/PD-L1 antibody arm, when compared with the other two treatments.

The benefit of efficacy should be balanced against the risk of toxic effects. However, an analysis of toxicity profile could not be conducted as the data of adverse events from each WT subgroup was not available. Even so, within the scope of overall patients with advanced NSCLC, treatment-related grade 3-4 toxic effects in PD-1/PD-L1 arm occurred less frequently than in chemotherapy arm in randomized trials (7%-26.6% vs 35%-55%) [24-27, 34, 35]. Though immune-related adverse events such as pneumonitis can occur and may be severe, most events are low grade and can be improved/resolved with drug holding/imunosuppression [36]. These results suggested that, for pre-treated WT EGFR patients, PD-1/PD-L1 antibody could be a preferable treatment choice over chemotherapy and TKI.

Our analysis also showed that chemotherapy was associated with longer OS than EGFR-TKIs in indirect comparison. But this benefit was not identified in our standard pairwise comparison (*p* = 0.27) or previous meta-analyses [20, 21]. There were probably two reasons for this issue. First, in almost all trials comparing EGFR-TKIs with chemotherapy, patients were allowed to receive postprogression crossover therapy and subsequent treatments. These factors were difficult to balance and confounded OS result. Second, except three modern trials only recruited WT EGFR patients [17, 19, 32], a majority of trials were designed for unselected patients and WT EGFR patients represented only a subgroup that included small sample size, which might bias outcome. With the method of Bayesian indirect comparison, we integrated indirect evidence of 4 trials accruing 1990 patients and head-to-head evidence of 8 trials with 1375 patients to enhance the statistical power, identifying a 12% OS improvement in chemotherapy arm. In the only trial that forbade crossover therapy and only included WT EGFR patients—TAILOR, treatment of chemotherapy was associated with a longer median OS than erlotinib (adjusted HR 0.73, 95% CI 0.53-1.00; *p* = 0.05) [17]. Furthermore, in Japanese randomized phase III DELTA trial, the researchers noticed a trend toward better OS in chemotherapy arm than in erlotinib arm for the WT EGFR patients who did not received subsequent chemotherapy [18]. These reports suggested that the OS difference was diminished by mixed treatments and supported our finding. In addition, we also noticed that patients in seond-line therapy tended to have a longer survival when treated with chemotherapy than TKI, though the *p* value did not reach a significance threshold (HR 0.85, 95% CI 0.71-1.01, *p* = 0.06). Consistent with previous studies, a 25% PFS prolongation in chemotherapy was also detected in our study.

Erlotinib is currently recommanded as a potential option for WT EGFR patinets in second-line [13]. Yet, our subgroup analysis showed that, chemotherapy was associated with better PFS and comparable OS when compared with erlotinib (Figure 5), which suggested that erlotinib might not be active enough even in second- or third-line therapy.

Given the milder toxicity profile of TKI than chemotherapy that has been demonstrated in included trials, TKI may only be considered for the patients who are not candidates for chemotherapy (such as the ones with bad performance status).

Additionally, economic burden should also be considered. Reported as the cost-effectiveness studies, for the lifetime per advanced NSCLC patient, deocetaxel is comparable to erlotinob in second/third-line therapy when estimated with the data from large randomized trials or national health systems [37-41]. In contrast, the current cost with nivolumab and pembrolizumab were about 3.5 and 7 times higher than deocetaxel in second- or third-line therapy, respectively. Despite of the much higher prices, a trade-off was found between prolonged survival and quality-adjusted-life years, and increasd cost. This indicated nivolumab and pembrolizumab could be considered as a cost-effective option [42, 43].

Potential limitations need to be taken into account. First, like most of the published meta-analysis, our analysis is based on the summary data from published literature rather than individual patient data, which limit the detail that can be captured regarding prognostic factors. Therefore, our findings need to be considered as average effects. Second, the WT EGFR patients included in most individual trials represented a subgroup that accounted for only a small portion of the enrolled patients (12%-52%), which might bias outcome. This is because the role of EGFR mutation as a predictive marker for TKI had not been established when these trials were initially designed in the early days. Though these trials were overall low risk, relavant results should be cautiously interpreted. Third, to date, no head-to-head randomized trials have compared the effecacy of PD-1/PD-L1 to EGFR-TKI for WT EGFR patients, leading to an unclosed loop of evidence in this study. Although we found no significant interstudy heterogeneity in all direct comprisons (up to 27.9% of the *I*^*2*^ value), the conclusion for indirect comparisons may be further confirmed by randomized controlled trials.To the best of our knowledge, this is the first meta-analysis comparing immune checkpoint inhibitor, EGFR-TKI and chemotherapy for advanced NSCLC patients with WT EGFR tumors in second- or third-line therapy. We showed that PD-1/PD-L1 antibody appeared to be the most efficacious treatment, which was followed by chemotherapy. EGFR-TKI was worse than chemotherapy. These findings suggested that, for pre-treated WT EGFR patients, PD-1/PD-L1 antibody can be a preferable option. For the ones who are not candidates for PD-1/PD-L1 antibody therapy, chemotherapy is preferred. TKI may be only considered for the ones who have bad performance status.

## MATERIALS AND METHODS

### Search strategy and selection criteria

Two reviewers (W.D. and C.D.) separately underwent systematic searches of PubMed, Cochrane databases and EMBASE, combining key terms “non-small cell lung”, “gefitinib”, “erlotinib”, “nivolumab”, “pembrolizuma” and “atezolizumab” until the end of January 2017, with no date and language restriction (see full search strategy in Supplementary Materials). The trials meeting the following criteria were included: (i) randomized controlled trials (RCTs) enrolling for pre-treated patients with advanced NSCLC, defined as unresectable locally advanced, metastatic or recurred disease (stage IIIB or IV). (ii) RCTs investigating two or more treatments among standard chemotherapy, first-generation EGFR-TKI and PD-1/PD-L1. (iii) RCTs reporting hazard ratios (HRs) with 95% confidence intervals (CIs) for OS and/or PFS, or data to estimate these in patients with WT EGFR tumors. The trials that assessed maintenance strategy, used combination agents of TKI with chemotherapy or anti-angiogenic agents, or only contained patients with EGFR-positive tumors were excluded. Placebo-controlled trials were also excluded. The bibliographies of relevant reviews and meta-analyses were also manually examined to identify additional trials. If multiple articles covered the same trial population, the trial with the most updated and complete data was included.

### Data extraction

Two reviewers (W.D. and C.D.) separately abstracted data in a predefined format, including trial acronym, trial design, main entry criteria, line of treatment, dominant ethnicity, method of EGFR mutation detection, interventional and control treatments, dosing schedule, number of patients randomized to each arm on an intention-to-treat (ITT) basis, demographic and clinical data (age, ethnicity, histology), outcome results in patients with WT EGFR tumors and duration of follow-up. The primary endpoint in this meta-analysis is OS, defined as the time interval from randomization to death from any cause. The second pre-specified endpoint is PFS, defined as the time interval from randomization to earliest occurrence of documented disease progression or death from any cause. The HRs, which represent the most appropriate metric for time-to-event outcomes, were directly extracted from each trial or estimated by the method of Tierney and colleagues if they were not explicitly provided [44]. Imformative meta-analyses were also used to acquire unreported data. Unadjusted HRs were preferred in this meta-analysis, given the adjusted ones were likely to adjust with different covariates from trial to trial, potentially impeding analysis. Two reviewers (W.D. and C.D.) separately assess the risk of bias of included trials with the Cochrane Collaboration method [45]. Data and bias discrepancies were discussed by all authors to reach consensus.

### Statistical analysis

Multiple treatment comparisons were built by WinBUGS 1.4.3 (MRC Biostatistics Unit, Cambridge, UK), allowing for the combination of direct and indirect evidence into a combined overall point estimate. HRs were pooled by posterior means with corresponding 95% credible intervals (CrIs), which are the Bayesian analog of the 95 % confidence intervals (CIs) [46]. Non-informative uniform and normal prior distributions were used to fit the model, yielding 50,000 iterations with a burn-in number of 10,000 iterations and a thin interval of 50 to obtain the posterior distributions of the model parameters [47]. Then the deviance information criterion (DIC) statistics were used to compare the two effect-models: a lower DIC value indicated a better model fit, and the corresponding results were chosen for summary estimation [48]. Convergence of iterations was evaluated by Gelman-Rubin-Brooks statistic [49]. The probability of each treatment in the ranking was evaluated based on its posterior probabilities, which depended on counting the proportion of iterations in the Markov chain of HR ranking in the treatments.

Standard pairwise comparisons were built with STATA 12.0 (STATA Crop., College Station, TX, USA). The interstudy heterogeneity was measured by *I*^2^ index [50], The pooled HRs from indirect comparisons were compared with corresponding HRs from pairwise comparisons to assess whether there was inconsistency. The effect of prespecified study-level characteristics including dominant ethnicity (white or Asian), line of treatment (first line or higher line), the specific TKI used (erlotinib vs gefitinib) and method of EGFR mutation detection (direct sequencing or more sensitive methods) was assessed by subgroup analysis and meta-regression. Statistical tests were two-sided and used a significance threshold of *p* < 0.05.

## SUPPLEMENTARY MATERIALS



## Abbreviations

ARMS: amplification-refractory mutation system
CIs: confidence intervals
CrIs: credible intervals
CT: Chemotherapy
DIC: deviance information criterion
EGFR: epidermal growth factor receptor
FISH: fluorescence in situ hybridization
IQR: interquartile range
HR: hazard ratio
MS: mass spectrometry
NR: not reported
NSCLC: non-small-cell lung cancer
PD-1: programmed death-1
PCR: polymerase chain reaction
PD-L1: programmed death-ligand 1
TKI: tyrosine kinase inhibitor
RCT: randomized controlled trials
WT: wild type
