# Supplementary material for: Which treatment is preferred for advanced non-small-cell lung cancer with wild-type epidermal growth factor receptor in second-line therapy? A meta-analysis comparing immune checkpoint inhibitor, tyrosine kinase inhibitor and chemotherapy
Source: Oncotarget. 2017 Aug 16;8(39):66491–503. doi: 10.18632/oncotarget.20281 (PMC5630430; doi:10.18632/oncotarget.20281)
Supplement: Supplementary file 1 [file oncotarget-08-66491-s001.pdf]

Which treatment is preferred for advanced non-small-cell lung cancer with wild-type epidermal growth factor receptor in second-line therapy? A meta-analysis comparing immune checkpoint inhibitor, tyrosine kinase inhibitor and chemotherapy

SUPPLEMENTARY MATERIALS

PubMed search strategy: 917 records
#1 gefitinib [tiab] OR iressa [tiab] OR ZD1839 [tiab]
#2 erlotinib [tiab] OR tarceva [tiab] OR OSI774 [tiab]
#3 #1 OR #2
#4 anti-PD1 [tiab]
#5 Nivolumab [tiab] OR ONO-4538 [tiab] OR MDX-1106 [tiab] OR BMS-936558 [tiab]
#6 Pembrolizumab [tiab] OR Keytruda [tiab] OR lambrolizumab [tiab] OR MK-3475 [tiab]
#7 anti-PDL1 [tiab]
#8 Atezolizumab [tiab] OR Tecentriq [tiab] OR MPDL3280A [tiab] OR RG-7446 [tiab]
#9 #3 OR #4 OR #5 OR #6 OR #7 OR #8
#10 carcinoma, non-small-cell lung [Mh] OR (NSCLC [All Fields] OR (non-small cell [tiab] AND lung [tiab]
#11 (randomized controlled trial [pt] OR controlled clinical trial [pt] OR randomized [tiab] OR placebo [tiab] OR clinical trials as topic [mesh: noexp] OR randomly [tiab] OR trial [ti]) NOT (animals [mh] NOT humans [mh])
#12 #9 AND #10 AND #11

Embase search strategy: 1401 records
#1 (gefitinib OR iressa OR ZD1839):ab,ti
#2 (erlotinib OR tarceva OR OSI774):ab,ti
#3 #1 OR #2
#4 (Nivolumab OR ONO-4538 OR MDX-1106 OR BMS-936558):ab,ti
#5 (Pembrolizumab OR Keytruda OR lambrolizumab OR MK-3475):ab,ti
#6 (Atezolizumab OR Tecentriq OR MPDL3280A OR RG-7446):ab,ti
#7 #3 OR #4 OR #5 OR #6
#8 (carcinoma, nonsmall-cell lung OR non-small cell lung cancer OR nsclc) AND human/de
#9 (random* OR blind* OR placebo OR 'meta analysis'):ab,ti
#10 #7 AND #8 AND #9

Cochrane database search strategy: 658 records
#1 (gefitinib OR iressa OR ZD1839):ti,ab,kw
#2 (erlotinib OR tarceva OR OSI774):ti,ab,kw
#3 #1 OR #2
#4 (Nivolumab OR ONO-4538 OR MDX-1106 OR BMS-936558):ti,ab,kw
#5 (Pembrolizumab OR Keytruda OR lambrolizumab OR MK-3475):ti,ab,kw
#6 (Atezolizumab OR Tecentriq OR MPDL3280A OR RG-7446):ti,ab,kw
#7 #3 OR #4 OR #5 OR #6
#8 (carcinoma, nonsmall-cell lung OR non-small cell lung cancer OR nsclc):ti,ab,kw
#11 trials
